# 

**Published:** 1893-04-08

**Authors:** 


CONTENTS.

Prescribing in the Dark - . . . . . . . . . . . - 17

Around the Hospitals - . . . . . . . . . . - 18

Medical History from the Earliest Times. By E. T. WITHINGTON, M.A., M.B.Oxon. XLIX.—Similars, Sympathy, and Signatures - . . . . . . . . . . - 19

Contemporary Theory and Research . . . . . - 20

Diet in Disease. By Mrs. ERNEST HART. XIX.—Indigestion - 21

Gleanings in Science and Medicine - . . . . . . - 22

Annotations.—A Curious Survival at Tunbridge Wells—The Beginnings of Insanity—Rags and Reason - . . . . . . - 23

Notes and News - . . . . . . . . . . - 24

The London Poor and Their Medical Needs . . . . - 31

Scraps and Gleanings - . . . . . . . . . - 32

The Hospital Clinic—

THE ROYAL ORTHOPÆDIC HOSPITAL.—The Treatment of Flat Foot, Bow Legs, and Knock Knee - . . . . . . . - 25

BRISTOL GENERAL HOSPITAL.—Treatment of Fractured Patella - 26

ST. MARY'S HOSPITAL.—The Treatment of Acute Rheumatism - 27

NEW DRUGS, APPLIANCES, AND THINGS MEDICAL.—A New Laundry Soap - . . . . . . . . . . . . - 28

The Institutional Workshop—

HOSPITAL CONSTRUCTION.—New Hospital for Children at Leipzig 29

PRACTICAL DEPARTMENTS.—Smoke-Consuming Stoves - . . - 30

CONSTRUCTION NOTES - . . . . . . . . . . - 30

Book World of Medicine and Science- . . . . . viii

The Student of Medicine—Appointments, Vacancies . . x

EXTRA SUPPLEMENT.—THE NURSING MIRROR.

News from the Nursing World.—A Royal Inspector—Attributes to Aim At.—How Not to Nourish Babies—A Good Beginning—Progress Opposed—Novel Training Schools—What will they Cost?—How to Spread Infection—Woman's Share—Voluntary Support—An Unfulfilled Promise—Nurses in Wales—Economy at Melbourne—Good News for Kandy—Nurses at Berlin—"The Hospital" Endowed Bed - . . . xi

Management of Consumption. IX.—The Feeding of Consumptives - . . . . . . . . . . . . xiii

Jottings from the Punjab - . . . . . . . . xiv

Where to Go - . . . . . . . . . . . . xiv

Nursing at Natal.—II. A Private Nurse's Opinion . . xiv

Notes and Queries . . . . . . . . . xv

Test Questions. — Given to Nurses of the State Hospital, Utica, New York, prior to their Examination . xvi

Hints for Nurses - . . . . . . . . . . xvi

Minor Appointments- . . . . . . . . . xvi

Everybody's Opinion.—India—Co operation—Management of Consumption: Explanation — Hints from America — The Young Nurse - . . . . . . . . . xvii

For Reading to the Sick.—Resurgam - . . . . . xvii

Appointments—Wants and Workers - . . . . . xviii

The Muses' Looking-Glass - . . . . . . . xviii

The Book World for Women and Nurses . . . . xix

